# Adverse Pregnancy Outcomes and Incident Heart Failure in the Women’s Health Initiative

**DOI:** 10.1001/jamanetworkopen.2021.38071

**Published:** 2021-12-09

**Authors:** Aleksander L. Hansen, Marc Meller Søndergaard, Mark A. Hlatky, Eric Vittinghof, Gregory Nah, Marcia L. Stefanick, JoAnn E. Manson, Leslie V. Farland, Gretchen L. Wells, Morgana Mongraw-Chaffin, Erica P. Gunderson, Linda Van Horn, Robert A. Wild, Buyun Liu, Aladdin H. Shadyab, Matthew A. Allison, Simin Liu, Charles B. Eaton, Michael C. Honigberg, Nisha I. Parikh

**Affiliations:** 1 Steno Diabetes Center Odense, Odense University Hospital, Odense, Denmark; 2 Aalborg University School of Medicine and Health, Aalborg, Denmark; 3 Department of Health Research and Policy, Stanford University School of Medicine, Stanford, California; 4 Department of Epidemiology and Biostatistics, University of California San Francisco School of Medicine, San Francisco; 5 Department of Medicine, Division of Cardiology, University of California, San Francisco; 6 Department of Preventive Medicine, Brigham and Women’s Hospital, Harvard Medical School, Boston, Massachusetts; 7 Mel and Enid Zuckerman College of Public Health, University of Arizona, Tucson; 8 College of Medicine, University of Kentucky, Lexington; 9 Department of Epidemiology and Prevention, Wake Forest University School of Medicine, Winston-Salem, North Carolina; 10 Lifecourse Epidemiology of Diabetes and Heart Disease in Women and Youth Division of Research, Kaiser Permanente Northern California, Oakland; 11 Feinberg School of Medicine, Northwestern University, Chicago, Illinois; 12 Department of Biostatistics and Epidemiology, Oklahoma University Health Sciences Center, Oklahoma City; 13 Department of Obstetrics and Gynecology, Oklahoma University Health Sciences Center, Oklahoma City; 14 Department of Epidemiology, University of Iowa, Iowa City; 15 School of Medicine, University of California San Diego, La Jolla; 16 Department of Epidemiology, Public Health Program, Brown University, Providence, Rhode Island; 17 Alpert Medical School, Brown University, Pawtucket, Rhode Island; 18 Cardiology Division, Massachusetts General Hospital, Boston; 19 Broad Institute of Harvard and Massachusetts Institute of Technology, Cambridge

## Abstract

**Importance:**

Some prior evidence suggests that adverse pregnancy outcomes (APOs) may be associated with heart failure (HF). Identifying unique factors associated with the risk of HF and studying HF subtypes are important next steps.

**Objective:**

To investigate the association of APOs with incident HF overall and stratified by HF subtype (preserved vs reduced ejection fraction) among postmenopausal women in the Women’s Health Initiative (WHI).

**Design, Setting, and Participants:**

In 2017, an APO history survey was administered in the WHI study, a large multiethnic cohort of postmenopausal women. The associations of 5 APOs (gestational diabetes, hypertensive disorders of pregnancy [HDP], low birth weight, high birth weight, and preterm delivery) with incident adjudicated HF were analyzed. In this cohort study, the association of each APO with HF was assessed using logistic regression models and with HF subtypes using multinomial regression, adjusting for age, sociodemographic characteristics, smoking, randomization status, reproductive history, and other APOs. Data analysis was performed from January 2020 to September 2021.

**Exposures:**

APOs (gestational diabetes, HDP, low birth weight, high birth weight, and preterm delivery).

**Main Outcomes and Measures:**

All confirmed cases of women hospitalized with HF and HF subtype were adjudicated by trained physicians using standardized methods.

**Results:**

Of 10 292 women (median [IQR] age, 60 [55-64] years), 3185 (31.0%) reported 1 or more APO and 336 (3.3%) had a diagnosis of HF. Women with a history of any APO had a higher prevalence of hypertension, diabetes, coronary heart disease, or smoking. Of the APOs studied, only HDP was significantly associated with HF with a fully adjusted odds ratio (OR) of 1.75 (95% CI, 1.22-2.50), and with HF with preserved ejection fraction in fully adjusted models (OR, 2.06; 95% CI, 1.29-3.27). In mediation analyses, hypertension explained 24% (95% CI, 12%-73%), coronary heart disease 23% (95% CI, 11%-68%), and body mass index 20% (95% CI, 10%-64%) of the association between HDP and HF.

**Conclusions and Relevance:**

In this large cohort of postmenopausal women, HDP was independently associated with incident HF, particularly HF with preserved ejection fraction, and this association was mediated by subsequent hypertension, coronary heart disease, and obesity. These findings suggest that monitoring and modifying these factors early in women presenting with HDP may be associated with reduced long-term risk of HF.

## Key Points

**Question:**

Are adverse pregnancy outcomes independently associated with the development of heart failure among postmenopausal women?

**Findings:**

In this cohort study including 10 292 Women’s Health Initiative participants, hypertensive disorders of pregnancy were independently associated with incident heart failure, particularly heart failure with preserved ejection fraction, in postmenopausal women.

**Meaning:**

These findings suggest that hypertensive disorders of pregnancy are sex-specific factors associated with risk of heart failure, particularly heart failure with preserved ejection fraction.

## Teaser

This cohort study investigates the association of adverse pregnancy outcomes with incident heart failure overall and stratified by heart failure subtype among postmenopausal women in the Women’s Health Initiative.

## Introduction

Women account for most cases of heart failure (HF) with preserved ejection fraction (HFpEF).^1^ Approximately 85% of US women experience pregnancy and childbirth, and up to 30% of pregnancies are complicated by 1 or more adverse pregnancy outcomes (APOs).^2^ Several APOs have been associated with a higher risk of developing cardiovascular disease (CVD), including gestational diabetes (GD), hypertensive disorders of pregnancy (HDP), preterm delivery (PTD), low birth weight (LBW), and high birth weight (HBW).^3,4,5,6^

Prior studies^7,8,9,10^ suggest that preeclampsia, gestational hypertension, and GD may be associated with an increased risk of developing HF. However, prior studies have neither jointly considered the associations of multiple APOs with HF nor distinguished between HFpEF and HF with reduced ejection fraction (HFrEF) because of limited HF phenotyping.^7,8,11,12^ In addition, mediators of these associations have not been robustly explored to date. Given the availability of both reproductive data and adjudicated HF outcomes, the Women’s Health Initiative (WHI) is a unique resource to test the individual and joint associations between APOs and HF.

## Methods

### Study Population

The WHI is a longitudinal study of ethnically diverse postmenopausal women aged 50 to 79 years at entry, recruited from 40 US clinical centers between 1993 and 1998, and followed prospectively since enrollment for multiple outcomes. Details of recruitment, baseline questionnaires, and examinations performed have been described elsewhere.^13,14^ Briefly, women participated in 1 or more of 3 clinical trials (of hormone therapy, dietary modification, and calcium or vitamin D supplementation) or enrolled in an observational study.

This cohort study follows the Strengthening the Reporting of Observational Studies in Epidemiology (STROBE) reporting guidelines and was approved by the University of California San Francisco institutional review board. All participants gave written informed consent to participate in WHI and its extension study.

**Figure 1.**
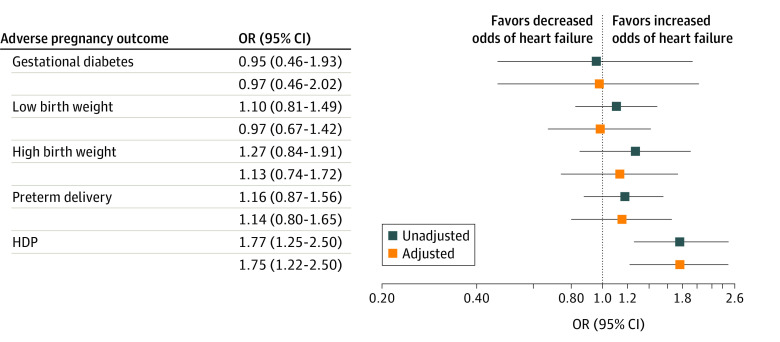
Forest Plot of Associations of Adverse Pregnancy Outcomes (APOs) With Heart Failure Figure shows association between individual APOs and heart failure. Each line displays the odds ratio (OR) and its 95% CIs from the comparison of yes responses with no response, based on logistic regression. The top line for each APO shows the OR for the subsequent APO from the unadjusted model. The bottom line of each APO shows the OR adjusted for age, pack-years of smoking, randomization status, race and ethnicity, education, income, number of live births, history of breastfeeding, age at first birth, menstrual cycle irregularity, age at menopause, oral contraceptive use, stillbirths, miscarriages, and subsequent APOs. HDP indicates hypertensive disorders of pregnancy.

Of the 161 808 women in the WHI cohort, a subset of 44 174 participants were included in the incident HF physician adjudication subcohort. The present study was based on women in the HF subcohort who completed the APO survey and were free of HF at entry into WHI (baseline) (Figure 1).

In 2017, a follow-up survey was sent to all surviving WHI participants.^3^ The survey included 6 questions on APOs during any pregnancy, with possible responses of no, yes, and do not know (eFigure 1 in the Supplement). APOs surveyed included GD, preeclampsia, gestational hypertension, PTD (<37 weeks gestation), LBW (<5.5 lb [2500 g]) or HBW (>9 lb 14 oz [4500 g]). More than 1 APO may have occurred in the same woman, but not necessarily during the same pregnancy. Any history of APO was defined as a participant reporting 1 or more APOs. Preeclampsia or eclampsia and gestational hypertension were combined into a single HDP variable because of substantial overlap in responses and similar point estimates with respect to their association with the outcome (ie, HF), as in prior published analyses.^3^

Participants in the HF subcohort were included in our study population if they completed the APO survey, had a history of pregnancy lasting for more than 6 months, and were alive and still participating in the WHI at the time of the survey. Nonresponders were defined as eligible WHI participants in the HF subcohort who did not answer the survey, had a history of pregnancy lasting for more than 6 months, and were alive and still participating in the WHI at the time of the survey (eFigure 2 in the Supplement).

### Outcomes

The primary outcome was development of an HF diagnosis during the WHI study follow-up period through 2018. Secondary outcomes included the development of HF subtypes HFrEF and HFpEF. All confirmed cases of HF hospitalization and patient-reported development of HF, angina, or CVD during hospitalization were adjudicated by trained physicians using standardized methods.^15,16,17^ Briefly, hospital records of suspected HF were abstracted to include evidence of new onset of symptoms, history of HF, general medical history, physical examination signs and symptoms, diagnostic tests, biomarkers (brain natriuretic peptide, N-terminal prohormone brain natriuretic peptide, and cardiac troponins), and medications. Physician adjudicators reviewed this information for evidence of HF. Subtypes of HF were classified as HFrEF for patients with EF less than 50% and as HFpEF for those with EF 50% or higher, consistent with American and European clinical practice guidelines^18,19,20^

### Covariates

Baseline characteristics were obtained by interviews and questionnaires at WHI study enrollment.^14,21^ Factors associated with risk included age at enrollment and pack-years of smoking. Reproductive factors included a history of breastfeeding (defined as breastfeeding for at least 1 month over the woman’s entire reproductive period), number of live births, stillbirths (defined as number of stillbirths from a pregnancy lasting ≥6 months), miscarriages (defined as number of spontaneous miscarriages), age at first term pregnancy, menstrual cycle irregularity, age at menopause, and history of oral contraceptive use. Sociodemographic factors included income (defined as annual household income), education level, and race and ethnicity determined from the questionnaire (non-Hispanic White, Black or African American, Hispanic or Latino, and other, which refers to American Indian or Alaskan Native, Asian or Pacific Islander [Chinese, Indo-Chinese, Korean, Japanese, Pacific Islander, Vietnamese], any other race or ethnicity, or not reported). Race and ethnicity were assessed in this study to ensure the generalizability of findings and to be able to detect any racial or ethnic disparities in associations. Randomization status indicates whether a participant has been randomized to 1 or more of the clinical trial components. Potential mediators included coronary heart disease (CHD) (defined by self-report of physician-diagnosed cardiovascular disease, or adjudicated first occurrence of clinical myocardial infarction, definite silent myocardial infarction, or coronary revascularization before or at the same time as HF outcome was diagnosed), hypertension (defined by self-report of physician-diagnosed hypertension, or systolic blood pressure ≥140 mm Hg or diastolic blood pressure ≥90 mm Hg at WHI study enrollment), diabetes (defined as not pregnancy related, self-reported physician diagnosis, or use of diabetes medication at study enrollment), and body mass index (BMI; defined as weight in kilograms divided by height in meters squared and measured by trained clinic staff at enrollment).

### Statistical Analysis

#### Multivariable Regression Analyses

Logistic regression tested the association of each APO with incident HF, adjusting for potential confounders. In this model, each APO was coded using 3 categories (yes, no, or do not know). The responses yes and do not know were compared with the reference category (no). In a secondary analyses, we tested whether results were changed in models in which the do not know responses were treated as missing and then imputed and a model where they were combined with the yes responses, as done previously^3^ (eTable 1 in the Supplement). For simplicity, we present these secondary analyses only in the supplement because findings were similar to those in the primary analysis.

The first model was unadjusted, and subsequent models were also adjusted for (1) age; (2) sociodemographic factors (race and ethnicity, education, and income), smoking, and randomization status; (3) other APOs and reproductive history (GD, HDP, LBW, HBW, PTD, live births, stillbirths, miscarriages, history of any or ever breastfeeding, age at first birth, menstrual cycle irregularity, age at menopause, and history of oral contraceptive use); and (4) a fully adjusted model including all covariates in the preceding models. In addition to examining HF overall, we performed multinomial regression on HF subtypes (no HF vs HFpEF vs HFrEF) using the same adjustment models. All models were estimated using multivariate imputation by chained equations, pooling results from 10 data sets using standard methods to capture the inflation of SEs by the imputation^22^ to create 10 data sets.

We used the mediation package in R^23^ to test potential mediation by hypertension, CHD, diabetes, and BMI for the association of APOs with HF. In brief, this approach uses nested models to estimate the proportion of the total adjusted association of an exposure explained by its indirect association via the mediator, with 95% CIs estimated using a nonparametric bootstrapping method. Each mediation analysis model was run using 1000 simulations. We applied full covariate adjustment to both the mediator and outcome model for consistency across mediation analyses.

#### Secondary Analyses

To make the questionnaire respondents more representative of the overall HF subcohort (eTable 2 in the Supplement) and to address potential survival bias, we performed sensitivity analysis using inverse probability of inclusion weights^24^ based on a logistic model for the association of baseline WHI covariates with inclusion in the questionnaire sample. We assessed modification of the associations of APOs with HF by race and ethnicity, age, BMI, hypertension, diabetes, CHD, history of breastfeeding, and by other APOs. Finally, we did a sensitivity analysis excluding women with CHD.

We considered 2-sided *P* < .05 to be statistically significant. Analyses were performed using R statistical software version 3.5.1 (R Project for Statistical Computing) and SAS Enterprise statistical software version 9.4 (SAS Institute). Data analysis was performed from January 2020 to September 2021.

## Results

### Adverse Pregnancy Outcomes: Characteristics

Of 44 174 women in the WHI HF subcohort, 27 204 had a history of pregnancy lasting for more than 6 months, were alive, and were still participating in the WHI at the time of the survey; 10 292 responded and formed the study population (eFigure 2 in the Supplement). The median (IQR) age of participants was 60 (55-64) years. In this study population, 3185 women (31.0%) reported a history of 1 or more APOs. The most frequently reported APO was PTD in 1509 women (14.7%), followed by LBW in 1424 (13.8%), HDP in 759 (7.4%), HBW in 644 (6.3%), and GD in 260 (2.5%) (Table 1). The most common combination of APOs in the study population was PTD and LBW and was reported by 732 women (7.1%).

**Table 1.** Distribution of APO Survey Answers

APO	APO survey answer, participants, No. (%)^a^			
	No	Yes	Do not know	Missing
Gestational diabetes	9699 (94.2)	260 (2.5)	265 (2.6)	68 (0.7)
Low birth weight	8667 (84.2)	1424 (13.8)	107 (1.0)	94 (0.9)
High birth weight	9496 (92.3)	644 (6.3)	59 (0.6)	93 (0.9)
Preterm delivery	8409 (81.7)	1509 (14.7)	239 (2.3)	135 (1.3)
Hypertension disorder of pregnancy	8611 (83.7)	759 (7.4)	893 (8.7)	29 (0.3)
Any APO	6325 (61.5)	3185 (30.9)	782 (7.6)	0

Abbreviation: APO, adverse pregnancy outcome.

^a^ Women who answered yes to 1 or more APO are included in the any APO row. The number of women with any APO does not equal sum of yes responses, because women could have had more than 1 APO.

Baseline characteristics of women at entry into the WHI differed by the presence and type of APO (eTable 3 in the Supplement). Women with a history of any APO had a higher prevalence of hypertension, diabetes, CHD, smoking (>20 pack-years), and stillbirth; lower levels of education and household income; younger age at first birth and older age at menopause; higher BMI; were less likely to have reported a history of breastfeeding, miscarriage, and menstrual cycle irregularity; and reported fewer live births. HDP was more prevalent among Black women compared with White women. Hypertension at baseline was more prevalent among women who reported HDP (68 women [62%]) than among women reporting any APO (1339 women [42%]) or no APO (2066 women [33%]). Diabetes at baseline was more prevalent among women reporting previous GD (56 women [22%]) than among women reporting any other APO (168 women [5.3%]) or no APO (160 women [2.5%]). Women who developed HF during follow-up were more likely to have hypertension, diabetes, and a higher BMI than those who did not experience HF (Table 2).

**Table 2.** Baseline Characteristics According to HF Status

Variables	Participants, No. (%)			*P* value
	Total (N = 10 292)	Without HF (n = 9956)	With HF (n = 336)	
Age at enrollment, median (IQR), y	60 (55-64)	59 (55-64)	63 (58-68)	<.001
Pack-years of smoking				
0	5451 (54.6)	5277 (54.6)	174 (52.7)	Reference
<5	1619 (16.2)	1574 (16.3)	45 (13.6)	.46
5-20	1502 (15.0)	1453 (15.0)	49 (14.8)	.94
>20	1417 (14.2)	1355 (14.0)	62 (18.8)	.04
Body mass index, median (IQR)^a^	27.8 (24.7-32.0)	27.8 (24.6-31.9)	30 (26.0-36.4)	<.001
Randomization status				
No	1756 (17.1)	1709 (17.2)	47 (13.9)	Reference
Yes	8536 (82.9)	8244 (82.8)	292 (86.1)	.13
Race or ethnicity				
Black	2519 (24.5)	2446 (24.6)	73 (21.5)	.04
Hispanic	1147 (11.2)	1131 (11.4)	16 (4.7)	<.001
White	6387 (62.1)	6142 (61.8)	245 (72.3)	Reference
Other^b^	229 (2.2)	224 (2.3)	5 (1.5)	.25
Education				
Some college and above	6876 (67.3)	6665 (67.4)	211 (62.6)	Reference
High school and below	3344 (32.7)	3218 (32.6)	126 (37.4)	.09
Annual household income, $				
≥75 000	1765 (17.9)	1730 (18.1)	35 (10.7)	Reference
20 000-74 000	6727 (68.2)	6497 (68.1)	230 (70.3)	.003
<20 000	1370 (13.9)	1308 (13.7)	62 (19.0)	<.001
History of breastfeeding				
No	4302 (42.1)	4158 (42.0)	144 (43.1)	Reference
Yes	5926 (57.9)	5736 (58.0)	190 (56.9)	.70
Age at first birth, y				
<20	1938 (21.3)	1855 (21.1)	83 (27.7)	Reference
≥20	7154 (78.7)	6937 (78.9)	217 (72.3)	.007
Menstrual cycle irregularity				
No	796 (7.8)	770 (7.8)	26 (7.7)	Reference
Yes	8441 (82.5)	8159 (82.4)	282 (83.7)	.92
Sometimes regular, sometimes irregular	999 (9.8)	970 (9.8)	29 (8.6)	.76
Age at menopause, median (IQR), y	50 (45-52)	50 (45-52)	50 (45-52)	.92
Oral contraceptive				
No	4841 (47.0)	4647 (46.7)	194 (57.2)	Reference
Yes	5451 (53.0)	5306 (53.3)	145 (42.8)	<.001
Stillbirths				
0	9673 (95.2)	9351 (95.2)	322 (95.8)	Reference
1	485 (4.8)	471 (4.8)	14 (4.2)	.63
Miscarriages				
0	6753 (66.2)	6539 (66.3)	214 (63.9)	Reference
1	2296 (22.5)	2223 (22.5)	73 (21.8)	.99
≥2	1152 (11.3)	1104 (11.2)	48 (14.3)	.10
Live births				
0	148 (1.4)	145 (1.5)	3 (0.9)	Reference
1	1120 (10.9)	1085 (11.0)	35 (10.4)	.64
2	2724 (26.6)	2651 (26.8)	73 (21.7)	.52
3	2755 (26.9)	2672 (27.0)	83 (24.7)	.94
4	1803 (17.6)	1739 (17.6)	64 (19.0)	.61
≥5	1689 (16.5)	1611 (16.3)	78 (23.2)	.06
Hypertension				
No	6527 (63.4)	6365 (64.0)	162 (47.8)	Reference
Yes	3765 (36.6)	3588 (36.0)	177 (52.2)	<.001
Diabetes				
No	9928 (96.5)	9618 (96.6)	310 (91.4)	Reference
Yes	364 (3.5)	335 (3.4)	29 (8.6)	<.001
Coronary heart disease				
No	8588 (83.4)	8397 (84.4)	191 (56.3)	Reference
Yes	1704 (16.6)	1556 (15.6)	148 (43.7)	<.001

Abbreviation: HF, heart failure.

^a^ Body mass index is calculated as weight in kilograms divided by height in meters squared.

^b^ Other includes American Indian or Alaskan Native, Asian or Pacific Islander (ancestry is Chinese, Indo-Chinese, Korean, Japanese, Pacific Islander, Vietnamese), any other race or ethnicity, or not reported (from questionnaire).

### APOs, HF, and Mediation Analysis

**Figure 2.**
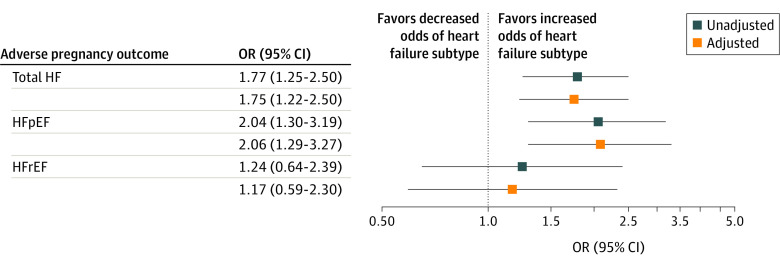
Forest Plot of Association of Hypertensive Disorders of Pregnancy (HDP) With Heart Failure (HF) Subtypes Association between individual adverse pregnancy outcomes (APOs) and HF subtypes, HF with preserved ejection fraction (HFpEF) and HF with reduced ejection fraction (HFrEF). Each line displays the odds ratio (OR) and its 95% CIs from the comparison of yes responses with no responses to HDP, based on multinomial logistic regression. The top line for each APO shows the OR for HDP from the unadjusted model. The bottom line of each APO shows the OR adjusted for age, pack-years of smoking, randomization status, race and ethnicity, education, income, number of live births, history of breastfeeding, age at first birth, menstrual cycle irregularity, age at menopause, oral contraceptive use, stillbirths, miscarriages, and subsequent APOs.

**Figure 3.**
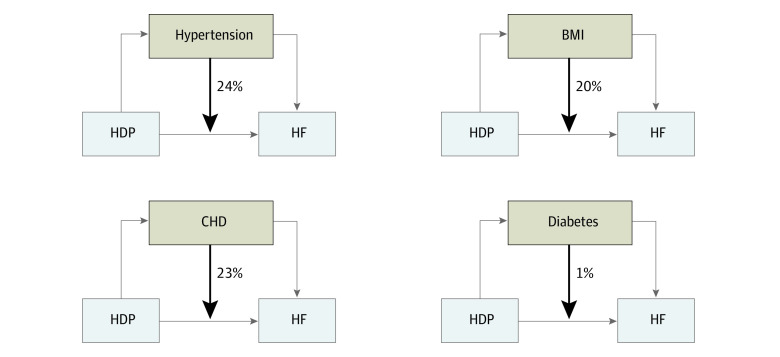
Mediation Analysis Each rectangle consists of a variable associated with the risk of hypertensive disorders of pregnancy (HDP), outcome variable heart failure (HF), and mediator (top rectangle). The arrow going from mediator down toward the arrow between risk variable and outcome variable shows the mediation of the association of HDP with HF. Our full model was applied to all mediation analysis for more consistent, parsimonious models across all mediation analyses. The association with diabetes was not significant. The mediation and outcome model were adjusted for age, pack-years of smoking, randomization status, race and ethnicity, education, income, number of live births, history of breastfeeding, age at first birth, menstrual cycle irregularity, age at menopause, oral contraceptive use, stillbirths, miscarriages, and subsequent adverse pregnancy outcomes. BMI indicates body mass index (calculated as weight in kilograms divided by height in meters squared); CHD, coronary heart disease.

Of our cohort of 10 292 participants, 336 (3.3%) had a diagnosis of HF, 180 (1.8%) had HFpEF, and 111 (1.1%) had HFrEF. Women with a history of APO had a higher rate of HF than those without a history of APO (121 women [3.8%] vs 184 women [2.9%]). Women with HDP had the highest rate of HF (39 women [5.1%]), and women with GD had the lowest rate of HF (8 women [3.1%]) (eFigure 3 in the Supplement). HDP was the only APO with a significant association with HF in univariate models, with an odds ratio (OR) of 1.77 (95% CI, 1.25 to 2.50). HDP remained significantly associated with HF after adjusting for age (OR, 1.87; 95% CI, 1.32 to 2.65), sociodemographic factors, smoking and randomization status (OR, 1.76; 95% CI, 1.25 to 2.50), other subsequent APOs and reproductive history (OR, 1.70; 95% CI, 1.19 to 2.42), and in a model adjusting for all these factors (OR, 1.75; 95% CI, 1.22 to 2.50) (Figure 1). In analyses of HF subtypes, only HDP was significantly associated with HFpEF in a fully adjusted model (OR, 2.06; 95% CI, 1.29 to 3.27), but not with HFrEF (OR, 1.17; 95% CI, 0.59 to 2.30) (Figure 2). In mediation analysis, hypertension explained 24% (95% CI, 12% to 73%) of the association of HDP with HF, BMI explained 20% (95% CI, 10% to 64%), diabetes explained 1% (95% CI, −3.6% to 7.3%), and CHD explained 23% (95% CI, 11% to 68%) (Figure 3).

### Other Secondary Analyses

All results from the analysis were virtually unchanged in the complete case analysis (eTable 1 in the Supplement) and in models with inverse probability of inclusion weights (eTable 4 in the Supplement). We found no significant modification of the association of HDP with HF by covariates including other APOs. Upon excluding women with CHD, the association between HDP and HF was similar (OR, 1.67; 95% CI, 1.01-2.76).

## Discussion

In this large cohort study of postmenopausal women, a history of HDP was independently associated with a 1.75-fold odds of developing subsequent heart failure. This association was significant after adjustment for multiple confounding factors, including other APOs, without evidence of modification by sociodemographic or reproductive factors or comorbidities. HDP was significantly associated with developing HFpEF, but not HFrEF, among women in late midlife. Furthermore, the association of HDP with HF was partially mediated by hypertension, BMI, and CHD. Our findings highlight that a subset of women with HDP will not develop hypertension before developing HF.

Several mechanisms may explain the association between HDP and HF. They share several risk factors, such as hypertension and obesity, which underlie this association.^7^ HDP is associated with the development of hypertension,^25^ and having both is associated with persistent left ventricular remodeling.^26^ A meta-analysis revealed that HDP and HFpEF share several biomarkers, including immune activation, myocardial stress, and autonomic function.^27^ Women with a history of HDP have persistent structural differences in the heart and microvasculature more than 25 years after their pregnancy.^28,29^ Women with a history of HDP have increased echocardiographic diastolic parameters, including left ventricular mass index, increased relative wall thickness, lower impaired relaxation (ie, lower transmitral Doppler E/A ratio), and higher left ventricular filling pressure (ie, E/e′ ratio) in comparison with those with a history of normotensive pregnancies, all of which are associated with HF.^28,30,31^ Women with HDP also have impaired coronary flow reserve, which is a key feature of HFpEF.^32^ Persistence of these changes may contribute preferentially to risk of HFpEF (vs HFrEF).^33^

APOs may also be associated with the development of CVD risk factors themselves, which could mediate associations between APOs and future CVD.^34^ Whether an APO uncovers a predisposition to CVD, exacerbates a preexisting subclinical condition, or initiates a pathway that results in CVD is unclear, although recent data may suggest all may be true.^33,34^

Our mediation analysis showed large but incomplete mediations of the associations from hypertension (24%), BMI (20%), and CHD (23%). These findings align with known factors associated with the risk of HFpEF.^35^ However, this also implies that CVD-related factors may not fully explain the association between HDP and HF. Two previous studies^4,36^ found that hypertension mediated 49% of the association of HDP with HF and cardiomyopathy.

HFpEF disproportionately affects elderly women, and hypertension is an important factor associated with risk of HFpEF among women.^37^ Furthermore, HFrEF is less common in women than in men.^38^ Our findings that HDP is independently associated with HFpEF and that hypertension is a mediator of the association between HDP and HF suggest that both hypertension and novel pathways likely explain the HDP and HFpEF association we found in our study. It has been unclear whether some APOs are more closely related to HF and CVD risk, because different APOs may share some elements of underlying pathophysiology.^2,39^ Our findings show that only a history of HDP was independently associated with HF in postmenopausal women and suggests that this APO merits particular scrutiny in future epidemiological and mechanistic studies.

Although prior studies demonstrated an association between GD and HF,^8^ we did not confirm this association. The women in our study, however, reported a relatively low prevalence of GD (2.5%, vs the current US prevalence of 8%).^40^ This difference may reflect evolving practices for GD screening, which was not widely and routinely implemented in the US until the early 1980s, after many women in the present data set would have completed their pregnancies, as well as an increasing prevalence of factors associated with the risk of GD among reproductive-aged women (eg, obesity or family history of diabetes).^41^ Therefore, we may have both underestimated the prevalence of GD in our study and had a cohort of women less likely to have GD. Contemporary studies assessing the association between GD and HF would be less likely to have these limitations.^42,43^

### Clinical Implications

History of HDP represents an opportunity for early, aggressive, preventive interventions for HF and other CVD, possibly before development of the traditional risk factors (hypertension, diabetes, and obesity). In fact, the recent Postnatal Enalapril to Improve Cardiovascular Function Following Preterm Preeclampsia Study^44^ showed improved diastolic function and left ventricular remodeling after 6 months of postnatal enalapril treatment for women with preterm preeclampsia. The concept of the fourth trimester has been introduced by the obstetric-gynecologic community to highlight the need to retain focus on optimizing maternal health beyond the standard 6-week postdelivery window to enable more targeted and aggressive risk factor modification in women with APOs,^45^ with lifestyle interventions, earlier monitoring, and tighter control of traditional risk factors, such as hypertension, diabetes, and obesity. Long-term studies are needed to assess to what extent earlier cardiovascular prevention techniques will prove effective in women with a history of HDP.^46^

### Strengths and Limitations

Strengths of this study include the unique availability of comprehensive baseline characteristics, reproductive history, and adjudicated HF outcomes, which allowed us to comprehensively evaluate the association between APOs and HF outcomes with long-term follow-up. This study also has limitations. Survivorship bias among the included sample resulted from participants needing to survive until the APO survey in 2017, be free of HF at baseline, and women with HDP (especially severe and recurrent preeclampsia) having increased mortality compared with other women.^12,47^ Therefore, our estimates would have been biased toward the null rather than leading to spuriously high estimates. However, our sensitivity analysis using inverse probability of inclusion weights demonstrated that the findings were similar. The WHI cohort is known to be representative regarding race and ethnicity,^48^ but our substudy had fewer racial and ethnic minority women than the overall WHI. The obstetric records were unavailable to us for validating the APO information, providing more specific APO phenotyping such as HDP severity. Therefore, we cannot confirm or deny the possibility that recall bias affected our results. WHI did not have information on prepregnancy risk factors such as BMI or gestational weight gain. We do not have information on when this cohort of postmenopausal women had their deliveries, which presents a potential recall bias, and women’s ability to recall APOs has only been validated previously in short-term studies, but not in long-term studies.^49^ We were unable to differentiate whether women had multiple APOs in the same pregnancy or in recurrent pregnancies, nor did we have information on size for gestational age.

## Conclusions

In this study, a history of HDP was associated with HF, particularly HFpEF, among postmenopausal women, independently of conventional HF risk factors, other APOs, and sociodemographic and reproductive factors. Close clinical monitoring of women with a history of HDP may provide opportunities for early prevention of HF and other CVD. Hypertension, BMI, and CHD played partial, mediating roles in the associations demonstrated. Further research is needed to better understand the potential mechanisms that link HDP with later development of HF. Dedicated studies are needed to establish effective interventions to mitigate long-term risk of HF and other CVD in women with APOs.

## Notes

Supplement. **eFigure 1.** Flow Diagram for the Selection of Study Participants **eFigure 2.** 2017 WHI Survey on Adverse Pregnancy Outcomes, Dental Health, Memory, and Changes in Mental Functioning (Form 158) **eFigure 3.** Bar Graph Heart Failure According to Answers in the Adverse Pregnancy Outcome Survey **eTable 1.** Comparison of Complete Case Analysis and Main Analysis With Multiple Imputations **eTable 2.** Baseline Characteristics of Eligible Nonresponders and Responders of the Adverse Pregnancy Outcome Survey in the WHI Heart Failure Cohort **eTable 3.** Baseline Characteristics According to the Different Adverse Pregnancy Outcomes **eTable 4.** Inverse Probability of Inclusion Weights

## References

[1] Borlaug BA, Redfield MM. Diastolic and systolic heart failure are distinct phenotypes within the heart failure spectrum. Circulation. 2011;123(18):2006-2013. doi:10.1161/CIRCULATIONAHA.110.954388 21555723 PMC3420141

[2] Rich-Edwards JW, Fraser A, Lawlor DA, Catov JM. Pregnancy characteristics and women’s future cardiovascular health: an underused opportunity to improve women’s health? Epidemiol Rev. 2014;36:57-70. doi:10.1093/epirev/mxt006 24025350 PMC3873841

[3] Søndergaard MM, Hlatky MA, Stefanick ML, . Association of adverse pregnancy outcomes with risk of atherosclerotic cardiovascular disease in postmenopausal women. JAMA Cardiol. 2020;5(12):1390-1398. doi:10.1001/jamacardio.2020.4097 32936228 PMC7495331

[4] Honigberg MC, Zekavat SM, Aragam K, . Long-term cardiovascular risk in women with hypertension during pregnancy. J Am Coll Cardiol. 2019;74(22):2743-2754. doi:10.1016/j.jacc.2019.09.052 31727424 PMC6981240

[5] Morken NH, Halland F, DeRoo LA, Wilcox AJ, Skjaerven R. Offspring birthweight by gestational age and parental cardiovascular mortality: a population-based cohort study. BJOG. 2018;125(3):336-341. doi:10.1111/1471-0528.14522 28165208 PMC5821431

[6] Parikh NI, Gonzalez JM, Anderson CAM, . Adverse pregnancy outcomes and cardiovascular disease risk: unique opportunities for cardiovascular disease prevention in women—a scientific statement from the American Heart Association. Circulation. 2021;143(18):e902-e916. doi:10.1161/CIR.0000000000000961 33779213

[7] Leon LJ, McCarthy FP, Direk K, . Preeclampsia and cardiovascular disease in a large UK pregnancy cohort of linked electronic health records: a CALIBER study. Circulation. 2019;140(13):1050-1060. doi:10.1161/CIRCULATIONAHA.118.038080 31545680

[8] McKenzie-Sampson S, Paradis G, Healy-Profitós J, St-Pierre F, Auger N. Gestational diabetes and risk of cardiovascular disease up to 25 years after pregnancy: a retrospective cohort study. Acta Diabetol. 2018;55(4):315-322. doi:10.1007/s00592-017-1099-2 29327149

[9] Lo CCW, Lo ACQ, Leow SH, . Future cardiovascular disease risk for women with gestational hypertension: a systematic review and meta-analysis. J Am Heart Assoc. 2020;9(13):e013991. doi:10.1161/JAHA.119.013991 32578465 PMC7670531

[10] Wu P, Haththotuwa R, Kwok CS, . Preeclampsia and future cardiovascular health: a systematic review and meta-analysis. Circ Cardiovasc Qual Outcomes. 2017;10(2):e003497. doi:10.1161/CIRCOUTCOMES.116.003497 28228456

[11] Bolijn R, Onland-Moret NC, Asselbergs FW, van der Schouw YT. Reproductive factors in relation to heart failure in women: a systematic review. Maturitas. 2017;106:57-72. doi:10.1016/j.maturitas.2017.09.004 29150167

[12] Bellamy L, Casas JP, Hingorani AD, Williams DJ. Pre-eclampsia and risk of cardiovascular disease and cancer in later life: systematic review and meta-analysis. BMJ. 2007;335(7627):974. doi:10.1136/bmj.39335.385301.BE 17975258 PMC2072042

[13] Langer RD, White E, Lewis CE, Kotchen JM, Hendrix SL, Trevisan M. The Women’s Health Initiative Observational Study: baseline characteristics of participants and reliability of baseline measures. Ann Epidemiol. 2003;13(9)(suppl):S107-S121. doi:10.1016/S1047-2797(03)00047-4 14575943

[14] The Women’s Health Initiative Study Group. Design of the Women’s Health Initiative clinical trial and observational study. Control Clin Trials. 1998;19(1):61-109. doi:10.1016/S0197-2456(97)00078-0 9492970

[15] Heckbert SR, Kooperberg C, Safford MM, . Comparison of self-report, hospital discharge codes, and adjudication of cardiovascular events in the Women’s Health Initiative. Am J Epidemiol. 2004;160(12):1152-1158. doi:10.1093/aje/kwh314 15583367

[16] Liu L, Klein L, Eaton C, . Menopausal hormone therapy and risks of first hospitalized heart failure and its subtypes during the intervention and extended postintervention follow-up of the Women’s Health Initiative randomized trials. J Card Fail. 2020;26(1):2-12. doi:10.1016/j.cardfail.2019.09.006 31536806

[17] The Women’s Health Initiative. WHI heart failure data summary, 2014. Accessed November 20, 2020. https://www.whi.org/dataset/559

[18] Zakeri R, Cowie MR. Heart failure with preserved ejection fraction: controversies, challenges and future directions. Heart. 2018;104(5):377-384. doi:10.1136/heartjnl-2016-310790 29305560

[19] Yancy CW, Jessup M, Bozkurt B, ; American College of Cardiology Foundation/American Heart Association Task Force on Practice Guidelines. 2013 ACCF/AHA guideline for the management of heart failure: a report of the American College of Cardiology Foundation/American Heart Association Task Force on practice guidelines. Circulation. 2013;128(16):e240-e327. doi:10.1161/CIR.0b013e31829e8776 23741058

[20] Ponikowski P, Voors AA, Anker SD, ; ESC Scientific Document Group. 2016 ESC Guidelines for the diagnosis and treatment of acute and chronic heart failure: the task force for the diagnosis and treatment of acute and chronic heart failure of the European Society of Cardiology (ESC) developed with the special contribution of the Heart Failure Association (HFA) of the ESC. Eur Heart J. 2016;37(27):2129-2200. doi:10.1093/eurheartj/ehw128 27206819

[21] Curb JD, McTiernan A, Heckbert SR, ; WHI Morbidity and Mortality Committee. Outcomes ascertainment and adjudication methods in the Women’s Health Initiative. Ann Epidemiol. 2003;13(9)(suppl):S122-S128. doi:10.1016/S1047-2797(03)00048-6 14575944

[22] van Buuren S, Groothuis-Oudshoorn K. mice: Multivariate imputation by chained equations in R. J Stat Softw. 2011;45(3):1-67. doi:10.18637/jss.v045.i03

[23] Tingley D, Yamamoto T, Hirose K, Keele L, Imai K. mediation: R package for causal mediation analysis. J Stat Softw. 2014;59(5):1-38. doi:10.18637/jss.v059.i0526917999

[24] van der Wal WM, Geskus RB. ipw: An R package for inverse probability weighting. J Stat Softw. 2011;43(13):1-23. doi:10.18637/jss.v043.i13

[25] Vahedi FA, Gholizadeh L, Heydari M. Hypertensive disorders of pregnancy and risk of future cardiovascular disease in women. Nurs Womens Health. 2020;24(2):91-100. doi:10.1016/j.nwh.2020.02.001 32119830

[26] Countouris ME, Villanueva FS, Berlacher KL, Cavalcante JL, Parks WT, Catov JM. Association of hypertensive disorders of pregnancy with left ventricular remodeling later in life. J Am Coll Cardiol. 2021;77(8):1057-1068. doi:10.1016/j.jacc.2020.12.051 33632480 PMC10544734

[27] Alma LJ, Bokslag A, Maas AHEM, Franx A, Paulus WJ, de Groot CJM. Shared biomarkers between female diastolic heart failure and pre-eclampsia: a systematic review and meta-analysis. ESC Heart Fail. 2017;4(2):88-98. doi:10.1002/ehf2.12129 28451444 PMC5396047

[28] Boardman H, Lamata P, Lazdam M, . Variations in cardiovascular structure, function, and geometry in midlife associated with a history of hypertensive pregnancy. Hypertension. 2020;75(6):1542-1550. doi:10.1161/HYPERTENSIONAHA.119.14530 32306767 PMC7682801

[29] Scantlebury DC, Kane GC, Wiste HJ, . Left ventricular hypertrophy after hypertensive pregnancy disorders. Heart. 2015;101(19):1584-1590. doi:10.1136/heartjnl-2015-308098 26243788 PMC4568146

[30] Ciftci FC, Caliskan M, Ciftci O, . Impaired coronary microvascular function and increased intima-media thickness in preeclampsia. J Am Soc Hypertens. 2014;8(11):820-826. doi:10.1016/j.jash.2014.08.012 25455007

[31] Reddy M, Wright L, Rolnik DL, . Evaluation of cardiac function in women with a history of preeclampsia: a systematic review and meta-analysis. J Am Heart Assoc. 2019;8(22):e013545. doi:10.1161/JAHA.119.013545 31698969 PMC6915290

[32] Shah SJ, Lam CSP, Svedlund S, . Prevalence and correlates of coronary microvascular dysfunction in heart failure with preserved ejection fraction: PROMIS-HFpEF. Eur Heart J. 2018;39(37):3439-3450. doi:10.1093/eurheartj/ehy531 30165580 PMC6927847

[33] Parikh NI, Laria B, Nah G, . Cardiovascular disease-related pregnancy complications are associated with increased maternal levels and trajectories of cardiovascular disease biomarkers during and after pregnancy. J Womens Health (Larchmt). 2020;29(10):1283-1291. doi:10.1089/jwh.2018.7560 31934809 PMC7583330

[34] Stuart JJ, Tanz LJ, Missmer SA, . Hypertensive disorders of pregnancy and maternal cardiovascular disease risk factor development: an observational cohort study. Ann Intern Med. 2018;169(4):224-232. doi:10.7326/M17-2740 29971437 PMC6601621

[35] Zhai AB, Haddad H. The impact of obesity on heart failure. Curr Opin Cardiol. 2017;32(2):196-202. doi:10.1097/HCO.0000000000000370 28092289

[36] Behrens I, Basit S, Lykke JA, . Association between hypertensive disorders of pregnancy and later risk of cardiomyopathy. JAMA. 2016;315(10):1026-1033. doi:10.1001/jama.2016.1869 26954411

[37] Lam CS, Carson PE, Anand IS, . Sex differences in clinical characteristics and outcomes in elderly patients with heart failure and preserved ejection fraction: the Irbesartan in Heart Failure with Preserved Ejection Fraction (I-PRESERVE) trial. Circ Heart Fail. 2012;5(5):571-578. doi:10.1161/CIRCHEARTFAILURE.112.970061 22887722 PMC4768740

[38] Lam CSP, Arnott C, Beale AL, . Sex differences in heart failure. Eur Heart J. 2019;40(47):3859-3868c. doi:10.1093/eurheartj/ehz835 31800034

[39] Siddiqui N, Hladunewich M. Understanding the link between the placenta and future cardiovascular disease. Trends Cardiovasc Med. 2011;21(7):188-193. doi:10.1016/j.tcm.2012.05.008 22867697

[40] Deputy NP, Kim SY, Conrey EJ, Bullard KM. Prevalence and changes in preexisting diabetes and gestational diabetes among women who had a live birth: United States, 2012-2016. MMWR Morb Mortal Wkly Rep. 2018;67(43):1201-1207. doi:10.15585/mmwr.mm6743a2 30383743 PMC6319799

[41] Szmuilowicz ED, Josefson JL, Metzger BE. Gestational diabetes mellitus. Endocrinol Metab Clin North Am. 2019;48(3):479-493. doi:10.1016/j.ecl.2019.05.001 31345518 PMC7008467

[42] Albrecht SS, Kuklina EV, Bansil P, . Diabetes trends among delivery hospitalizations in the U.S., 1994-2004. Diabetes Care. 2010;33(4):768-773. doi:10.2337/dc09-1801 20067968 PMC2845025

[43] Carpenter MW, Coustan DR. Criteria for screening tests for gestational diabetes. Am J Obstet Gynecol. 1982;144(7):768-773. doi:10.1016/0002-9378(82)90349-0 7148898

[44] Ormesher L, Higson S, Luckie M, . Postnatal Enalapril to Improve Cardiovascular Function Following Preterm Preeclampsia (PICk-UP): a randomized double-blind placebo-controlled feasibility trial. Hypertension. 2020;76(6):1828-1837. doi:10.1161/HYPERTENSIONAHA.120.15875 33012200 PMC7610547

[45] American College of Obstetricians and Gynecologists. ACOG committee opinion No. 736: optimizing postpartum care. Obstet Gynecol. 2018;131(5):e140-e150. doi:10.1097/AOG.0000000000002633 29683911

[46] Seely EW, Tsigas E, Rich-Edwards JW. Preeclampsia and future cardiovascular disease in women: how good are the data and how can we manage our patients? Semin Perinatol. 2015;39(4):276-283. doi:10.1053/j.semperi.2015.05.006 26117165

[47] Theilen LH, Meeks H, Fraser A, Esplin MS, Smith KR, Varner MW. Long-term mortality risk and life expectancy following recurrent hypertensive disease of pregnancy. Am J Obstet Gynecol. 2018;219(1):107.e1-107.e6. doi:10.1016/j.ajog.2018.04.002 29630888 PMC6019643

[48] Hays J, Hunt JR, Hubbell FA, . The Women’s Health Initiative recruitment methods and results. Ann Epidemiol. 2003;13(9)(suppl):S18-S77. doi:10.1016/S1047-2797(03)00042-5 14575939

[49] Carter EB, Stuart JJ, Farland LV, . Pregnancy complications as markers for subsequent maternal cardiovascular disease: validation of a maternal recall questionnaire. J Womens Health (Larchmt). 2015;24(9):702-712. doi:10.1089/jwh.2014.4953 26061196 PMC4808284

