# Supplementary material for: Adverse Pregnancy Outcomes and Incident Heart Failure in the Women’s Health Initiative
Source: JAMA Netw Open. 2021 Dec 9;4(12):e2138071. doi: 10.1001/jamanetworkopen.2021.38071 (PMC8662370; doi:10.1001/jamanetworkopen.2021.38071)
Supplement: Supplement. — eFigure 1. Flow Diagram for the Selection of Study Participants eFigure 2. 2017 WHI Survey on Adverse Pregnancy Outcomes, Dental Health, Memory, and Changes in Mental Functioning (Form 158) eFigure 3. Bar Graph Heart Failure According to Answers in the Adverse Pregnancy Outcome Survey eTable 1. Comparison of Complete Case Analysis and Main Analysis With Multiple Imputations eTable 2. Baseline Characteristics of Eligible Nonresponders and Responders of the Adverse Pregnancy Outcome Survey in the WHI Heart Failure Cohort eTable 3. Baseline Characteristics According to the Different Adverse Pregnancy Outcomes eTable 4. Inverse Probability of Inclusion Weights [file jamanetwopen-e2138071-s001.pdf]

Supplemental Online Content

Hansen AL, Søndergaard MM, Hlatky MA, et al. Adverse pregnancy outcomes and incident heart failure in the Women's Health Initiative. *JAMA Netw Open*. 2021;4(12):e2138071. doi:10.1001/jamanetworkopen.2021.38071

eFigure 1. 2017 WHI Survey on Adverse Pregnancy Outcomes, Dental Health, Memory, and Changes in Mental Functioning (Form 158)

eFigure 2. Flow Diagram for the Selection of Study Participants

eFigure 3. Bar Graph Heart Failure According to Answers in the Adverse Pregnancy Outcome Survey

eTable 1. Comparison of Complete Case Analysis and Main Analysis With Multiple Imputations

eTable 2. Baseline Characteristics of Eligible Nonresponders and Responders of the Adverse Pregnancy Outcome Survey in the WHI Heart Failure Cohort

eTable 3. Baseline Characteristics According to the Different Adverse Pregnancy Outcomes

eTable 4. Inverse Probability of Inclusion Weights

This supplemental material has been provided by the authors to give readers additional information about their work.

© 2021 Hansen AL et al. *JAMA Network Open.*

eFigure 1: 2017 WHI Survey on Adverse Pregnancy Outcomes, Dental Health, Memory, and Changes in Mental Functioning

Ver. 1

Form 158
Supplemental Questionnaire 2017

This questionnaire has questions about your experiences. Please answer each question as honestly as you can. No one will see your answers except for the scientists and staff at WHI. (Use a pencil or blue or black pen only.)

– Barcode ID Label –

1. Are you willing to provide your email address as an additional way for us to contact you?
   - Yes ____________________________________
     Print your email address on the line above.
   - No

These questions are about pregnancies.

2. Have you ever been pregnant?
   - No ⟶ **Go to question 3.**
   - Yes

2.1 Were any of your babies born 3 weeks early or sooner?
No / Yes / Don't know

2.2 During any of your pregnancies, did you have preeclampsia (toxemia, high blood pressure during and/or right after pregnancy also associated with protein in the urine) or eclampsia (preeclampsia AND seizures/convulsions)?
No / Yes / Don't know

2.3 During any of your pregnancies, did you have high blood pressure (gestational hypertension, pregnancy-induced hypertension) not related to preeclampsia?
No / Yes / Don't know

2.4 During any of your pregnancies, were you told you had gestational diabetes or high blood sugar, or sugar in the urine?
No / Yes / Don't know

2.5 Have you ever given birth to a baby that weighed less than 5 pounds, 8 ounces (less than 2,500 grams)?
No / Yes / Don't know

2.6 Have you ever given birth to a baby that weighed more than 9 pounds, 14 ounces (more than 4,500 grams)?
No / Yes / Don't know

These questions are about dental health.

3. How would you describe the condition of your mouth and teeth?
   Excellent / Very good / Good / Fair / Poor

4. During the past 3 years, how often have you gone to the dentist or dental hygienist for routine check-ups or cleanings?
   Never / Once or less per year / Twice or more per year / As needed

PLEASE MAKE NO MARKS IN THIS AREA

SERIAL #

R:\DOC\EXT2021\FORMS\F158V1.DOC 1/2017 [illegible] Page 1 of 2 GPO U.S. GOVERNMENT PRINTING OFFICE:[illegible]

WHI **Form 158 – Supplemental Questionnaire 2017** Ver. 1

5. Has a dentist or dental hygienist ever told you that you had periodontal or gum disease?
   No / Yes / Don't know

6. Have you lost all of your permanent teeth, both upper and lower?
   No / Yes

These questions are about memory and changes in mental functioning.

7. Do you feel like your memory is becoming worse?
   No / Yes, but this does not worry me / Yes, and this worries me

This next set of questions asks you to rate any change in your abilities, daily functioning and activities. Fill in the circle for each question that best fits your current ability level compared to 5 years ago.

**Select the one best choice for each item and** ***please do not skip any questions.***	No change	Minimal change	Some change	Clearly noticeable change	Much worse
7.1 Recalling information when I really try:	○1	○2	○3	○4	○5
7.2 Remembering names and faces of new people I meet:	○1	○2	○3	○4	○5
7.3 Remembering things that have happened recently:	○1	○2	○3	○4	○5
7.4 Recalling conversations a few days later:	○1	○2	○3	○4	○5
7.5 Remembering where things are usually kept:	○1	○2	○3	○4	○5
7.6 Remembering new information told to me:	○1	○2	○3	○4	○5
7.7 Remembering where I placed familiar objects:	○1	○2	○3	○4	○5
7.8 Remembering what I intended to do:	○1	○2	○3	○4	○5
7.9 Remembering names of family members and friends:	○1	○2	○3	○4	○5
7.10 Remembering without notes and reminders:	○1	○2	○3	○4	○5
7.11 People who know me would find that my memory is:	○1	○2	○3	○4	○5
7.12 Remembering things compared to my age group:	○1	○2	○3	○4	○5

This question is about your birth name.

8. U.S. census records have important historical information about the environment during your childhood, which may impact health. We would like to link your name to publicly available census records. Are you willing to provide your full birth name for this purpose?
   - Yes ⟶ Print your first, middle and last name as it appears **on your birth certificate**.
   - No

   First name ______ Middle name ______ Last name at birth (maiden name) ______

Thank you for taking the time to complete this questionnaire.

R:\DOC\EXT2021\FORMS\F158V1.DOC 1/2017 Page 2 of 2

(Form 158)

Legend: 2017 Women's Heath Initiative survey on adverse pregnancy outcomes, dental health, memory, and changes in mental functioning (Form 158). This survey was sent out to all living participants of the WHI in 2017 and are the basis of the adverse pregnancy outcomes information.

© 2021 Hansen AL et al. *JAMA Network Open.*

eFigure 2: Flow Diagram for the Selection of Study Participants

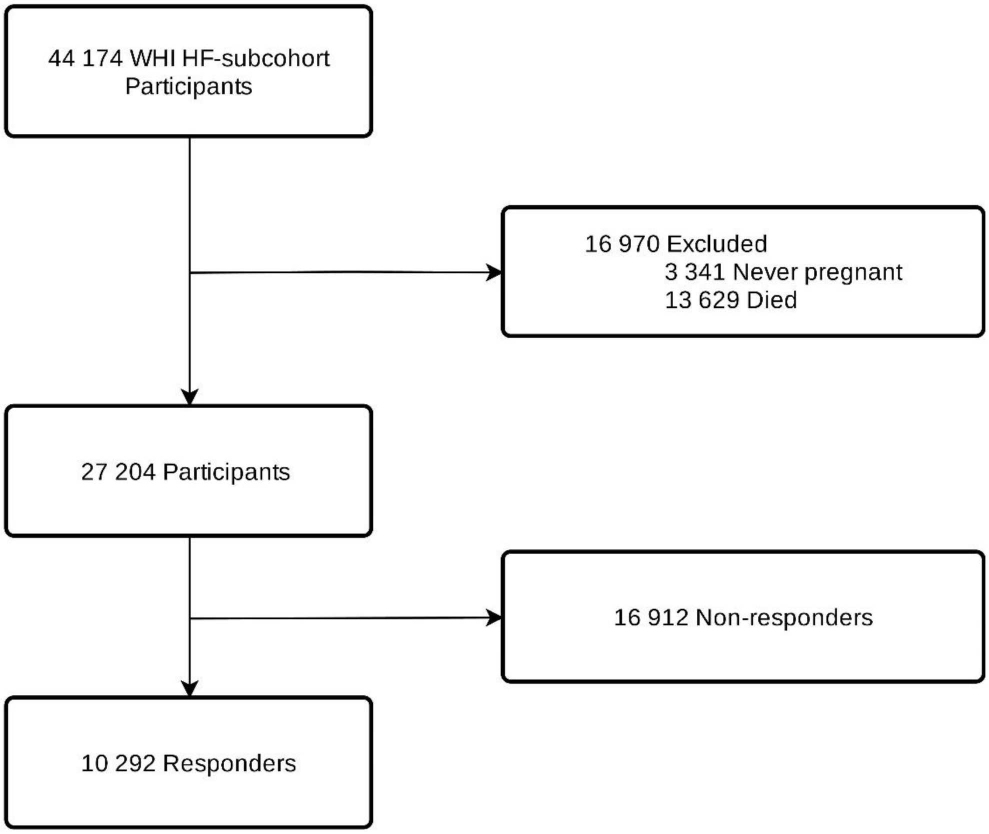

Legend: Flow diagram for the selection of study subjects from the Women's Health Initiative Study (WHI). Responders were defined as having completed the adverse pregnancy outcome survey, had a history of pregnancy lasting for more than six months and were alive and still participating in the WHI at the time of the survey. Non-responders were defined as eligible WHI participants in the heart failure subcohort who did not answer the survey, had a history of pregnancy lasting for more than six months, were alive and still participating in the WHI at the time of the survey.

© 2021 Hansen AL et al. *JAMA Network Open.*

eFigure 3: Bar Graph Heart Failure According to Answers in the Adverse Pregnancy Outcome Survey

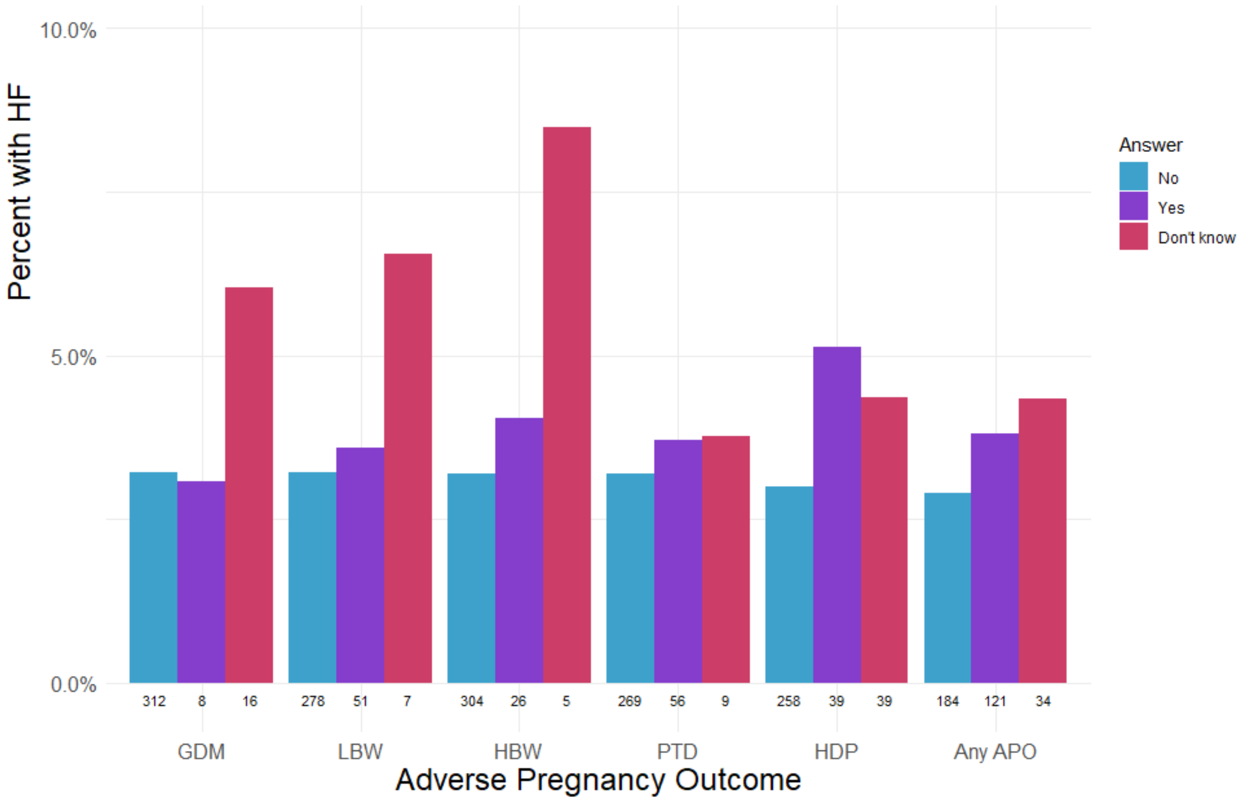

Legend: Bar graph displaying percentage heart failure according to answers in the APO survey (yes, no, do not know). Numbers underneath bars represent total number of answers for each category. Abbreviations: APO = Adverse pregnancy outcome; HF = heart failure; GDM = gestational diabetes mellitus; LBW = low birth weight; HBW = high birth weight; PTD = preterm delivery; HDP = hypertensive disorders of pregnancy

© 2021 Hansen AL et al. *JAMA Network Open.*

eTable 1: Comparison of Complete Case Analysis and Main Analysis With Multiple Imputations.

Main model – "Yes" vs "No"		
	OR [95% CI]	
**APO**	**Complete case analysis**	**Multiple imputations**
Gestational diabetes	1.11 [0.47, 2.29]	0.97 [0.46, 2.02]
Low birth weight	1.08 [0.69, 1.65]	0.97 [0.68, 1.42]
High birth weight	1.11 [1.16, 1.78]	1.13 [0.74, 1.72]
Preterm delivery	1.14 [0.75, 1.72]	1.14 [0.80, 1.65]
Hypertensive disorders of pregnancy	1.99 [1.32, 2.91]	1.75 [1.22, 2.50]

Main model – "Don't Know" vs "No"		
	OR [95% CI]	
**APO**	**Complete case analysis**	**Multiple imputations**
Gestational diabetes	0.74 [0.26, 1.73]	1.37 [0.75, 2.50]
Low birth weight	1.88 [0.59, 4.80]	1.33 [0.54, 3.24]
High birth weight	2.56 [0.55, 8.51]	1.80 [0.62, 5.19]
Preterm delivery	0.53 [0.15, 1.36]	0.81 [0.39, 1.67]
Hypertensive disorders of pregnancy	0.92 [0.53, 1.51]	1.06 [0.71, 1.58]

Secondary model 1 – "Don't know" set to missing		
	OR [95% CI]	
**APO**	**Complete case analysis**	**Multiple imputations**
Gestational diabetes	1.23 [0.50, 2.55]	1.00 [0.47, 2.11]
Low birth weight	1.15 [0.73, 1.79]	1.05 [0.72, 1.52]
High birth weight	1.23 [0.72, 1.99]	1.14 [0.75, 1.75]
Preterm delivery	1.16 [0.74, 1.77]	1.11 [0.77, 1.61]
Hypertensive disorders of pregnancy	1.88 [1.23, 2.79]	1.73 [1.22, 2.46]

Secondary model 2 – "Don't know" added to Yes		
	OR [95% CI]	
**APO**	**Complete case analysis**	**Multiple imputations**
Gestational diabetes	0.79 [0.41, 1.40]	1.07 [0.69, 1.70]
Low birth weight	1.23 [0.81, 1.82]	1.08 [0.76, 1.53]
High birth weight	1.18 [0.72, 1.85]	1.21 [0.82, 1.80]
Preterm delivery	0.97 [0.65, 1.42]	1.04 [0.75, 1.46]
Hypertensive disorders of pregnancy	1.43 [1.01, 1.99]	1.41 [1.05, 1.88]

© 2021 Hansen AL et al. *JAMA Network Open.*

Legend: Fully adjusted model used: Age, pack-years of smoking, randomization status, ethnicity, education, income, number of live births, history of breastfeeding, age at first birth, menstrual cycle irregularity, age at menopause, oral contraceptive use, still births, miscarriages and subsequent APOs. Main model APO answers coded as "No" (0), "Yes" (1), "Don't Know" (2). Secondary model 1: "Don't know" answers are set to missing and then used in the multiple imputations model. Secondary model 2: "don't know" answer is set to same value as "Yes" (1), i.e., added to "Yes" answer. Abbreviations: OR = Odds ratio; CI = confidence interval; APO = adverse pregnancy outcome

© 2021 Hansen AL et al. *JAMA Network Open.*

eTable 2: Baseline Characteristics of Eligible Nonresponders and Responders of the Adverse Pregnancy Outcome Survey in the WHI Heart Failure Cohort.

Variable	Level	Eligible non-responders n=16912 "No. (%)"	Responders n=10292 "No. (%)"	Total n=27204 "No. (%)"
**Age at enrollment (years)**	median [iqr]	60 [56, 66]	60 [55, 64]	60 [55, 65]
**Pack-years of smoking**	Non-smoker	9,012 (55.5)	5,451 (54.6)	14,463 (55.1)
	<5	2604 (16.0)	1619 (16.2)	4,223 (16.1)
	5-20	2382 (14.7)	1502 (15.0)	3,884 (14.8)
	>20	2253 (13.9)	1417 (14.2)	3,670 (14.0)
**Body Mass Index (kg/m^2)**	median [iqr]	29.1 [25.6, 33.4]	27.8 [24.7, 32.0]	28.6 [25.2, 32.9]
**Randomization status**	No	5,365 (31.7)	1,756 (17.1)	7,121 (26.2)
	Yes	11547 (68.3)	8536 (82.9)	20,083 (73.8)
**Ethnicity**	White	6,349 (37.6)	6,387 (62.1)	12,736 (46.9)
	Black	6587 (39.0)	2519 (24.5)	9,106 (33.5)
	Hispanic	3548 (21.0)	1147 (11.2)	4,695 (17.3)
	Other*	406 (2.4)	229 (2.2)	635 (2.3)
**Education**	High School & Below	9,132 (54.7)	6,876 (67.3)	16,008 (59.5)
	Some College & Above	7572 (45.3)	3344 (32.7)	10,916 (40.5)
**Income (USD)**	Below $20K	1,681 (10.8)	1,765 (17.9)	3,446 (13.6)
	$20K - $74K	9450 (61.0)	6727 (68.2)	16,177 (63.8)
	$75K Above	4371 (28.2)	1370 (13.9)	5,741 (22.6)
**History of breastfeeding**	No	7,655 (45.9)	4,302 (42.1)	11,957 (44.5)

© 2021 Hansen AL et al. *JAMA Network Open.*

	Yes	9007 (54.1)	5926 (57.9)	14,933 (55.5)
**Age at first birth (years)**	<20	3,804 (28.2)	1,938 (21.3)	5,742 (25.4)
	>20	9669 (71.8)	7154 (78.7)	16,823 (74.6)
**Menstrual cycle irregularity**	No	1,172 (7.0)	796 (7.8)	1,968 (7.3)
	Yes	14105 (84.2)	8441 (82.5)	22,546 (83.6)
	Sometimes regular, sometimes irregular	1472 (8.8)	999 (9.8)	2,471 (9.2)
**Age at menopause (years)**	median [iqr]	49 [43, 52]	50 [45, 52]	49 [45, 52]
**Oral contraceptive**	No	9,474 (56.0)	4,841 (47.0)	14,315 (52.6)
	Yes	7437 (44.0)	5451 (53.0)	12,888 (47.4)
**Still births**	No	15,273 (92.9)	9,673 (95.2)	24,946 (93.8)
	Yes	1165 (7.1)	485 (4.8)	1,650 (6.2)
**Miscarriages**	0	10,469 (63.2)	6,753 (66.2)	17,222 (64.3)
	1	3731 (22.5)	2296 (22.5)	6,027 (22.5)
	2+	2363 (14.3)	1152 (11.3)	3,515 (13.1)
**Live births**	0	852 (5.1)	148 (1.4)	1,000 (3.7)
	1	1918 (11.5)	1120 (10.9)	3,038 (11.3)
	2	4029 (24.1)	2724 (26.6)	6,753 (25.0)
	3	3899 (23.3)	2755 (26.9)	6,654 (24.7)
	4	2658 (15.9)	1803 (17.6)	4,461 (16.5)
	5+	3376 (20.2)	1689 (16.5)	5,065 (18.8)
**Hypertension**	No	9,156 (54.1)	6,527 (63.4)	15,683 (57.6)
	Yes	7756 (45.9)	3765 (36.6)	11,521 (42.4)

**Diabetes**	No	15,769 (93.2)	9,928 (96.5)	25,697 (94.5)
	Yes	1143 (6.8)	364 (3.5)	1,507 (5.5)
**CHD**	No	14,087 (83.3)	8,588 (83.4)	22,675 (83.4)
	Yes	2825 (16.7)	1704 (16.6)	4,529 (16.6)
**Heart failure**	No	16,550 (97.9)	9,953 (96.7)	26,503 (97.4)
	Yes	362 (2.1)	339 (3.3)	701 (2.6)
**Heart failure type**	No HF	16,602 (98.2)	9,998 (97.1)	26,600 (97.8)
	HFpEF	180 (1.1)	182 (1.8)	362 (1.3)
	HFrEF	130 (0.8)	112 (1.1)	242 (0.9)

Legend: Values are median [interquartile range] for continues variables and counts (%) for categorical variables. Responders were defined as having completed the adverse pregnancy outcome (APO) survey, had a history of pregnancy lasting for more than six months and were alive and still participating in the WHI at the time of the survey. Non-responders were defined as eligible Women's Health Initiative (WHI) participants in the heart failure (HF) subcohort who did not answer the survey, had a history of pregnancy lasting for more than six months, were alive and still participating in the WHI at the time of the survey. Abbreviations: CHD = coronary heart disease; iqr = interquartile range. *Other includes American Indian or Alaskan Native, Asian or Pacific Islander (ancestry is Chinese, Indo-Chinese, Korean, Japanese, Pacific Islander, Vietnamese), Other or not reported (from questionnaire)

© 2021 Hansen AL et al. *JAMA Network Open.*

eTable 3: Baseline Characteristics According to the Different Adverse Pregnancy Outcomes.

Variables	Levels	GDM ”No. (%)”	LBW ”No. (%)”	HBW ”No. (%)”	PTD ”No. (%)”	HDP ”No. (%)”	Any APO ”No. (%)”	No APO ”No. (%)”
**Age at enrollment (years)**	median [iqr]	57 [54, 61]	59 [55, 63]	60 [56, 65]	59 [55, 64]	59 [55.0, 63.5]	59 [55, 64]	59 [55, 64]
**Pack-years of smoking**	Non-smoker	149 (2.7)	696 (12.8)	375 (6.9)	753 (13.8)	408 (7.5)	1649 (30.3)	3405 (62.5)
	<5	45 (2.8)	209 (12.9)	114 (7.0)	235 (14.5)	122 (7.5)	510 (31.5)	985 (60.8)
	5-20	26 (1.7)	226 (15.0)	61 (4.1)	242 (16.1)	107 (7.1)	452 (30.1)	935 (62.3)
	>20	34 (2.4)	256 (18.1)	78 (5.5)	239 (16.9)	98 (6.9)	490 (34.6)	815 (57.5)
**Body Mass Index (kg/m^2)**	median [iqr]	30.9 [27.1, 34.9]	27.6 [24.5, 31.8]	30 [26.7, 34.3]	27.8 [24.5, 31.9]	29.6 [25.6, 34.4]	28.4 [25.1, 32.7]	27.5 [24.4, 31.6]
**Randomization Status**	No	49 (2.8)	258 (14.7)	83 (4.7)	249 (14.2)	145 (8.3)	538 (30.6)	1064 (60.6)
	Yes	211 (2.5)	1166 (13.7)	561 (6.6)	1260 (14.8)	614 (7.2)	2647 (31.0)	5261 (61.6)
**Ethnicity**	White	137 (2.1)	825 (12.9)	446 (7.0)	956 (15.0)	422 (6.6)	1954 (30.6)	4025 (63.0)
	Black	83 (3.3)	424 (16.8)	112 (4.4)	366 (14.5)	247 (9.8)	821 (32.6)	1453 (57.7)
	Hispanic	38 (3.3)	139 (12.1)	75 (6.5)	166 (14.5)	81 (7.1)	350 (30.5)	708 (61.7)
	Other*	2 (0.9)	36 (15.7)	10 (4.4)	21 (9.2)	9 (3.9)	59 (25.8)	133 (58.1)
**Education**	Some College & Above	164 (2.4)	933 (13.6)	393 (5.7)	1018 (14.8)	488 (7.1)	2090 (30.4)	4290 (62.4)
	High Sch & Below	95 (2.8)	481 (14.4)	245 (7.3)	482 (14.4)	268 (8.0)	1073 (32.1)	1989 (59.5)
**Income (USD)**	$75K Above	40 (2.3)	234 (13.3)	89 (5.0)	259 (14.7)	108 (6.1)	517 (29.3)	1147 (65.0)
	$20K - $74K	170 (2.5)	952 (14.2)	415 (6.2)	991 (14.7)	517 (7.7)	2098 (31.2)	4134 (61.5)
	Below $20K	44 (3.2)	187 (13.6)	118 (8.6)	196 (14.3)	115 (8.4)	455 (33.2)	778 (56.8)

© 2021 Hansen AL et al. *JAMA Network Open.*

**History of breastfeeding**	No	97 (2.3)	719 (16.7)	211 (4.9)	717 (16.7)	344 (8.0)	1415 (32.9)	2563 (59.6)
	Yes	161 (2.7)	692 (11.7)	428 (7.2)	782 (13.2)	410 (6.9)	1749 (29.5)	3722 (62.8)
**Age at first birth (years)**	<20	62 (3.2)	337 (17.4)	157 (8.1)	355 (18.3)	187 (9.6)	748 (38.6)	1054 (54.4)
	>20	177 (2.5)	966 (13.5)	418 (5.8)	1029 (14.4)	502 (7.0)	2148 (30.0)	4480 (62.6)
**Menstrual cycle irregularity**	No	22 (2.8)	115 (14.4)	47 (5.9)	131 (16.5)	74 (9.3)	269 (33.8)	460 (57.8)
	Yes	197 (2.3)	1143 (13.5)	526 (6.2)	1194 (14.1)	600 (7.1)	2547 (30.2)	5249 (62.2)
	Sometimes regular, sometimes irregular	40 (4.0)	159 (15.9)	67 (6.7)	176 (17.6)	80 (8.0)	356 (35.6)	579 (58.0)
**Age at menopause (years)**	median [iqr]	49 [44.5, 52.0]	50 [45, 52]	49 [45, 52]	50 [45, 52]	49 [44, 52]	50 [45, 52]	50 [46, 52]
**Oral contraceptive**	No	115 (2.4)	647 (13.4)	315 (6.5)	643 (13.3)	355 (7.3)	1448 (29.9)	2965 (61.2)
	Yes	145 (2.7)	777 (14.3)	329 (6.0)	866 (15.9)	404 (7.4)	1737 (31.9)	3360 (61.6)
**Still births**	No	228 (2.4)	1265 (13.1)	586 (6.1)	1327 (13.7)	685 (7.1)	2875 (29.7)	6077 (62.8)
	Yes	28 (5.8)	140 (28.9)	44 (9.1)	161 (33.2)	62 (12.8)	264 (54.4)	178 (36.7)
**Miscarriages**	0	142 (2.1)	817 (12.1)	394 (5.8)	840 (12.4)	482 (7.1)	1901 (28.2)	4365 (64.6)
	1	74 (3.2)	393 (17.1)	144 (6.3)	419 (18.2)	173 (7.5)	802 (34.9)	1314 (57.2)
	2+	41 (3.6)	206 (17.9)	97 (8.4)	242 (21.0)	97 (8.4)	457 (39.7)	589 (51.1)
**Live births**	0	1 (0.7)	18 (12.2)	5 (3.4)	31 (20.9)	6 (4.1)	41 (27.7)	80 (54.1)
	1	16 (1.4)	132 (11.8)	18 (1.6)	132 (11.8)	76 (6.8)	269 (24.0)	763 (68.1)
	2	71 (2.6)	291 (10.7)	80 (2.9)	326 (12.0)	205 (7.5)	702 (25.8)	1811 (66.5)
	3	64 (2.3)	387 (14.0)	191 (6.9)	427 (15.5)	188 (6.8)	869 (31.5)	1706 (61.9)

© 2021 Hansen AL et al. *JAMA Network Open.*

	4	44 (2.4)	257 (14.3)	123 (6.8)	274 (15.2)	139 (7.7)	580 (32.2)	1081 (60.0)
	5+	61 (3.6)	335 (19.8)	222 (13.1)	316 (18.7)	140 (8.3)	710 (42.0)	852 (50.4)
**Hypertension**	No	132 (2.0)	864 (13.2)	401 (6.1)	930 (14.2)	291 (4.5)	1846 (28.3)	4259 (65.3)
	Yes	128 (3.4)	560 (14.9)	243 (6.5)	579 (15.4)	468 (12.4)	1339 (35.6)	2066 (54.9)
**Diabetes**	No	204 (2.1)	1367 (13.8)	593 (6.0)	1452 (14.6)	717 (7.2)	3017 (30.4)	6165 (62.1)
	Yes	56 (15.4)	57 (15.7)	51 (14.0)	57 (15.7)	42 (11.5)	168 (46.2)	160 (44.0)
**CHD**	No	197 (2.3)	1146 (13.3)	533 (6.2)	1225 (14.3)	579 (6.7)	2577 (30.0)	5373 (62.6)
	Yes	63 (3.7)	278 (16.3)	111 (6.5)	284 (16.7)	180 (10.6)	608 (35.7)	952 (55.9)

Legend: Women who answered “No” to all adverse pregnancy outcome (APO) questions are included in the “No APO” column; women who answered “Yes” to one or more APO are included in the “Any APO” column. The different APO-columns refer to women who answered “Yes” (baseline characteristics of all APO survey answers for each APO are summarized in **supplemental table 1**. The number of women with “Any APO” does not equal sum of “Yes” responses, since women could have had more than one APO. Values are median [interquartile range] for continues variables and counts (%) for categorical variables. Abbreviations: CHD = coronary heart disease; iqr = interquartile range. GDM = Gestational diabetes mellitus; LBW = Low birth weight; HBW = High birth weight; PTD = Preterm delivery; HDP = Hypertensive disorders of pregnancy. *Other includes American Indian or Alaskan Native, Asian or Pacific Islander (ancestry is Chinese, Indo-Chinese, Korean, Japanese, Pacific Islander, Vietnamese), Other or not reported (from questionnaire)

© 2021 Hansen AL et al. *JAMA Network Open.*

eTable 4: Inverse Probability of Inclusion Weights

	**Inverse probability weighting OR [95% CI]**					
**APO**	**Main analysis**	**No trim**	**Trimmed at 25**	**Trimmed at 10**	**Trimmed at 5**	**Trimmed at 2**
Gestational diabetes	0.97 [0.46, 2.02]	0.77 [0.34, 1.73]	0.77 [0.34, 1.73]	0.77 [0.34, 1.73]	0.76 [0.33, 1.72]	0.81 [0.36, 1.79]
Low birth weight	0.97 [0.68, 1.42]	1.00 [0.63, 1.44]	0.96 [0.63, 1.44]	0.96 [0.63, 1.44]	0.96 [0.63, 1.44]	0.92 [0.61, 1.38]
High birth weight	1.13 [0.74, 1.72]	1.49 [0.97, 2.28]	1.49 [0.97, 2.28]	1.49 [0.97, 2.28]	1.49 [0.97, 2.28]	1.36 [0.88, 2.11]
Preterm delivery	1.14 [0.80, 1.65]	1.13 [0.75, 1.70]	1.13 [0.75, 1.70]	1.13 [0.75, 1.70]	1.13 [0.75, 1.70]	1.16 [0.78, 1.73]
Hypertensive disorders of pregnancy	1.75 [1.22, 2.50]	1.72 [1.17, 2.52]	1.72 [1.17, 2.53]	1.72 [1.17, 2.53]	1.72 [1.17, 2.53]	1.74 [1.19, 2.55]

Legend: The main analysis column represents the analysis presented as main finding in this paper i.e., association with HF. The second column represents values after including the weights from the inverse probability analysis. The four columns to the right represent values after including the weights trimmed at different cutoffs. All models are adjusted for the following: Age, pack-years of smoking, randomization status, ethnicity, education, income, number of live births, history of breastfeeding, age at first birth, menstrual cycle irregularity, age at menopause, oral contraceptive use, still births, miscarriages and subsequent APOs. Abbreviations: OR = Odds ratio; CI = confidence interval; APO = adverse pregnancy outcome; HF = heart failure.

© 2021 Hansen AL et al. *JAMA Network Open.*
